# Antioxidant and Anti-Inflammatory Activities of Methanol Extract of *Senna septemtrionalis* (Viv.) H.S. Irwin & Barneby Through Nrf2/HO-1-Mediated Inhibition of NF-κB Signaling in LPS-Stimulated Mouse Microglial Cells

**DOI:** 10.3390/ijms26051932

**Published:** 2025-02-24

**Authors:** Jae Sung Lim, Xiangying Li, Da Young Lee, Lulu Yao, Guijae Yoo, Yunyeong Kim, Sang Mi Eum, Young-Chang Cho, Somy Yoon, Su-Jin Park

**Affiliations:** 1 College of Pharmacy and Research Institute of Pharmaceutical Sciences, Chonnam National University, 77 Yongbong-ro, Gwangju 61186, Republic of Korea; dr.jslim7542@gmail.com (J.S.L.); lxyoung825@naver.com (X.L.); yl261213@gmail.com (L.Y.); oo0428@naver.com (Y.K.); yccho@jnu.ac.kr (Y.-C.C.); 2 R&D Center, CUOME BIO Co., Ltd., Sandan-gil, Hwasun-eup, Hwasun-gun 58141, Jeollanam-do, Republic of Korea; dlekdud0914@naver.com; 3 Korea Food Research Institute, 245, Nongsaengmyeong-ro, Iseo-myeon, Wanju-gun 55365, Jeollabuk-do, Republic of Korea; gjyoo@kfri.re.kr; 4 International Biological Material Research Center, Korea Research Institute of Bioscience & Biotechnology, 125 Gwahak-ro, Daejeon 34141, Republic of Korea; sangeum@gmail.com; 5 Functional Biomaterial Research Center, Korea Research Institute of Bioscience and Biotechnology, 181 Ipsin-gil, Jeongeup-si 56212, Republic of Korea

**Keywords:** *Senna septemtrionalis* (Viv.) H.S. Irwin & Barneby, antioxidant, anti-inflammatory, Nrf2, HO-1, NF-κB

## Abstract

Botanical extracts are recognized in traditional medicine for their therapeutic potential and safety standards. Botanical extracts are viable and sustainable alternatives to synthetic drugs, being essential in drug discovery for various diseases. *Senna septemtrionalis* (Viv.) H.S. Irwin & Barneby is a medical plant traditionally used to treat inflammation. However, its antioxidant and anti-inflammatory properties and the molecular pathways activated in microglial cells require further investigation. Therefore, this study examines the antioxidant and anti-inflammatory properties of *Senna septemtrionalis* (Viv.) H.S. Irwin & Barneby methanol extracts (SMEs) in lipopolysaccharide (LPS)-stimulated mouse microglial cells. SMEs significantly inhibit LPS-induced nitric oxide (NO) and proinflammatory cytokine production, which are mediated through the dephosphorylation of mitogen-activated protein kinases and inhibition of nuclear factor kappa B (NF-κB) translocation into the nucleus. Additionally, SME treatment upregulated the expression of nuclear factor erythroid 2-related factor 2 (Nrf2) and heme oxygenase (HO)-1, reducing oxidative stress, indicated by a decrease in reactive oxygen species and restoration of the total glutathione content in LPS-stimulated BV2 cells. The inhibitory effects of SMEs on inflammatory mediator production and NF-κB nuclear translocation were significantly reversed by Sn-protoporphyrin, a specific HO-1 inhibitor. These findings demonstrate that SME protects microglial cells by activating the Nrf2/HO-1 pathway and inhibiting NF-κB translocation.

## 1. Introduction

Microglia, the main immune cells in the central nervous system (CNS), are essential for maintaining neural homeostasis and overseeing neuronal function [1]. These cells are activated when external agents penetrate the blood–brain barrier (BBB), triggering immune responses to sustain the CNS’s stability [2,3]. Microglia release neurotoxic inflammatory mediators upon activation, including NO, proinflammatory cytokines such as IL-6, TNF-α, and IL-1β, and reactive oxygen species (ROS) [1]. However, prolonged microglial activation can lead to excessive secretion of these mediators, which is associated with the progression of neurodegenerative diseases, including Alzheimer’s disease (AD), Parkinson’s disease (PD), and multiple sclerosis (MS) [4,5,6,7].

ROS are essential signaling molecules, and their levels are tightly controlled under normal physiological conditions to preserve cellular homeostasis [8]. However, excessive ROS production, whether due to persistent stimulation or inadequate neutralization, can lead to cellular damage by targeting DNA, proteins, and lipids [9]. ROS act as secondary messengers in the microglial cellular inflammatory response, frequently leading to dysregulated immune activity [10]. Meanwhile, chronic overproduction of ROS has been associated with the development of numerous diseases, especially neurodegenerative disorders, where oxidative stress plays a crucial role in driving neuroinflammation [11,12]. For example, in cases of cerebral ischemia, oxidative stress initiates neuroinflammatory processes and activates both microglia and astrocytes, leading to the release of inflammatory mediators [13]. In Parkinson’s disease, oxidative stress caused by ROS plays a crucial role in activating microglia, which in turn leads to the degeneration of dopaminergic neurons [14,15]. Moreover, oxidative stress can disrupt mitochondrial function, thereby worsening neurodegenerative conditions, such as the development of amyloid-beta plaques in Alzheimer’s disease [16,17].

*Senna septemtrionalis* (*S*. *septemtrionalis*), commonly known as the arsenic bush, is a leafy shrub or small tree indigenous to Mexico and Central America [18]. This plant has been utilized in traditional medicine for many years due to its notable properties, including wound-healing, analgesic, and anti-inflammatory properties [19]. Recent studies have demonstrated that *S*. *septemtrionalis* extracts possess various therapeutic properties, including anti-inflammatory and anti-nociceptive activities in diuretic and neuropharmacological applications [20,21]. Phytochemical investigations have identified the presence of compounds such as anthraquinones, flavonoids, benzoic acids, and carboxylic acid in *S*. *septemtrionalis*, which are known for their roles in reducing inflammation and combating oxidative stress [21]. Although *S*. *septemtrionalis* has been widely used in traditional medicine, and recent research has highlighted its potential, the molecular mechanism underlying its ethnopharmacological anti-inflammatory and antioxidant effects remain unexplored.

Therefore, this study aimed to evaluate the anti-inflammatory and antioxidant activities of SMEs in the context of neuroinflammation. Furthermore, this research sought to uncover the specific molecular pathways through which SMEs exert their anti-inflammatory and antioxidant effects in LPS-stimulated BV2 microglial cells.

## 2. Results

### 2.1. Emodin Is a Major Component in SME

**Figure 1:**
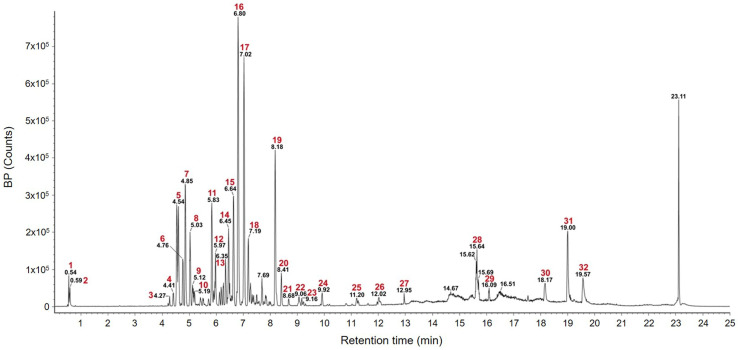
Total ion chromatogram (TIC) of the SME obtained using UPLC-Q-TOF-MS in negative ion mode with electrospray ionization (ESI/MS).

To assess the potential anti-inflammatory and antioxidant effects of SME, we first investigated the chemical composition using UPLC-Q-TOF-MS. The total ion chromatogram (TIC) (Figure 1) revealed a variety of compounds, including flavonoids (rutin, kaempferol, and complanatuside), triterpenoids (sericoside and ganoderenic acid F), saccharides (pentose), and the anthraquinone emodin (Table 1). A significant peak at a retention time of 6.8 minutes was identified as emodin, based on its parent ion at 269.0045 *m*/*z*. This identification was confirmed through the Waters Unifi Software Traditional Medicine Library (version: 1.9.4.053) (Waters Corporation, Milford, MA, USA) and corroborated by the existing literature [22,23]. Emodin, commonly found in various plant sources, including *S*. *septemtrionalis* [24], is known for its potent anti-inflammatory and antioxidant properties [25,26]. Additionally, other identified compounds, such as rutin, kaempferol, and genistein, are recognized for their anti-inflammatory and antioxidant activities [27,28,29]. These bioactive compounds collectively contribute to the observed bioactivity of *S*. *septemtrionalis*. Our findings emphasize the significant presence of emodin in the SME, highlighting its prominent role in overall bioactivity and reinforcing the relevance of further exploring the therapeutic potential of SMEs.

### 2.2. SME Has No Cytotoxic Effect on BV2 Cells

**Figure 2:**
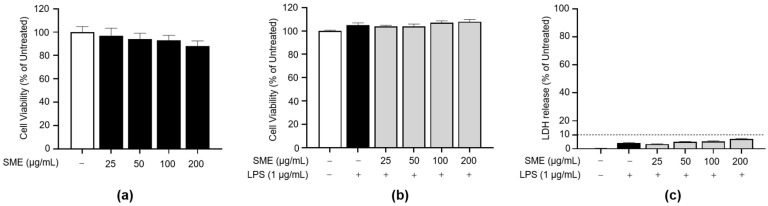
Effects of SME on cell viability of BV2 cells upon LPS stimulation. (**a**) BV2 cells were treated with serial concentrations of SME (25, 50, 100, and 200 μg/mL) for 24 h. (**b**,**c**) BV2 cells were pretreated with SME for 2 h, followed by stimulation with LPS (1 μg/mL) for 24 h. Cell viability in both (**a**,**b**) was assessed using the EZ-Cytox kit. (**c**) Cytotoxicity in BV2 cells pretreated with SME for 2 h and subsequently stimulated with LPS for 24 h was evaluated using an LDH assay. The dash line represents the threshold for cytotoxic effects.The data are expressed as the mean ± standard error of the mean (SEM) and are based on results from three separate experiments. Group comparisons were conducted using the Mann–Whitney U test for statistical analysis.

To determine the non-cytotoxic concentration range of SME, we evaluated its impact on BV2 cell viability over a range of concentrations (25–200 μg/mL) under both the presence and absence of LPS. As shown in Figure 2a,b, treatment with SME did not lead to a notable increase in cell death, suggesting that SME is not cytotoxic within the tested range. Additionally, LDH release, a marker of cellular damage, was assessed to validate these findings further. Consistent with the viability results, LDH release did not significantly increase following SME treatment at concentrations up to 200 μg/mL (Figure 2c). These data collectively indicate that SME is non-cytotoxic to BV2 cells at concentrations up to 200 μg/mL, providing a safe range for subsequent analyses of its antioxidant and anti-inflammatory effects.

### 2.3. SME Mitigates Inflammatory Responses in Microglial Cells Stimulated by LPS

**Figure 3:**
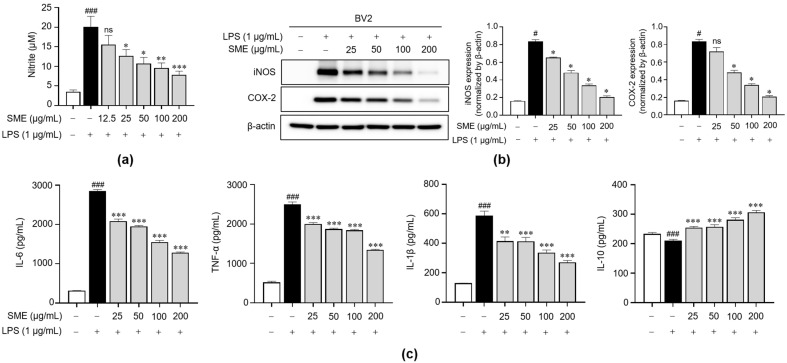
Impacts of SME on the inflammatory mediator production in LPS-stimulated BV2 cells. BV2 cells were pretreated with varying SME concentrations (25, 50, 100, and 200 μg/mL) for 2 h, followed by LPS (1 μg/mL) stimulation for 24 h. (**a**) NO levels in the cell supernatant were assessed using the Griess reagent, and the levels of NO secretion were determined using a standard curve of generated known nitrite concentrations. (**b**) COX-2 and iNOS protein expression were analyzed via western blot analysis, with β-actin serving as the loading control. The protein expression levels were normalized to β-actin, and they are depicted as a bar graph. (**c**) The IL-6, TNF-α, IL-1β, and IL-10 secretions were quantified using ELISA. The data are expressed as the mean ± standard error of the mean (SEM) and are based on results from three separate experiments. Group comparisons were conducted using the Mann–Whitney U test for statistical analysis, with significance thresholds set at *p* < 0.05, (^#^) *p* < 0.05, and (^###^) *p* < 0.001 compared with the untreated group and (*) *p* < 0.05, (**) *p* < 0.01, (***) *p* < 0.001, and not significant (ns) compared with the LPS-stimulated group.

To assess the impact of SME on neuroinflammation, we quantified the NO production, a key inflammatory mediator. Using the Griess reagent, we measured the nitrite levels, a stable metabolite of NO, to determine if SME could inhibit NO production. Our results revealed that SME significantly reduced nitrite production dose-dependently (Figure 3a). Given this reduction in NO, we further explored whether this effect was associated with changes in the expression of inducible nitric oxide synthase (iNOS), the enzyme which produces NO. Additionally, we examined the expression of cyclooxygenase-2 (COX-2), an enzyme which plays a role in producing the proinflammatory molecule PGE2, as both iNOS and COX-2 are known to be elevated in activated microglia during neuroinflammatory responses [42]. SME treatment led to a dose-dependent decrease in the protein levels of both iNOS and COX-2 in LPS-stimulated BV2 cells (Figure 3b). Subsequently, to gain deeper insight into the anti-inflammatory properties of SME, we assessed the expression levels of proinflammatory cytokines IL-1β, IL-6, and TNF-α, along with the anti-inflammatory cytokine IL-10. The ELISA results demonstrate that SME effectively reduced the production of proinflammatory cytokines in a dose-dependent fashion while simultaneously inducing the expression of IL-10 (Figure 3c). These findings indicate that SME provides neuroprotective effects by suppressing the production of NO, COX-2, and inflammatory cytokines while promoting the release of anti-inflammatory cytokines. These results suggest that SME confers neuroprotection by mitigating the production of inflammatory mediators.

### 2.4. SME Suppresses NF-κB Signaling in LPS-Stimulated BV2 Cells by Reducing p65 Subunit Translocation

**Figure 4:**
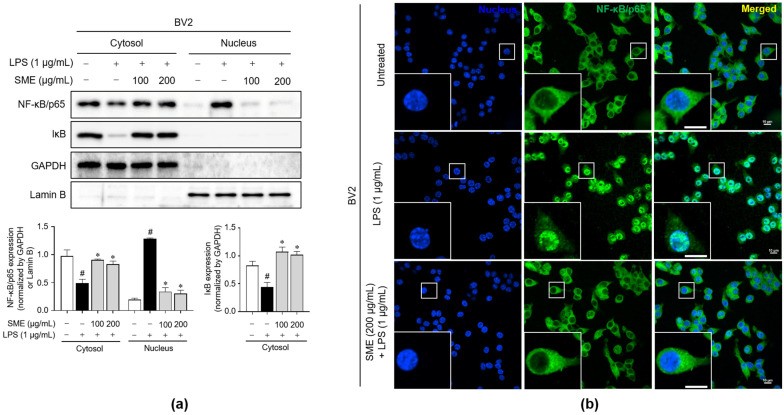
Impacts of SME on the NF-κB/p65 nuclear translocation in BV2 cells stimulated by LPS. BV2 cells were pretreated with SME (100 and 200 μg/mL) for 2 h, followed by LPS (1 μg/mL) stimulation for 15 min. (**a**) NF-κB/p65 and IκB protein expressions were analyzed in both cytosolic and nuclear fractions via western blot analysis, with GAPDH serving as the loading control for the cytosolic fraction. Meanwhile, lamin B was used as the nuclear loading control. The protein expression levels were normalized to GAPDH and lamin B, and they are depicted as a bar graph. The data are expressed as the mean ± standard error of the mean (SEM) and are based on results from three separate experiments. Group comparisons were conducted using the Mann–Whitney U test for statistical analysis, with significance thresholds set at *p* < 0.05, (^#^) *p* < 0.05 compared with the untreated group, and (*) *p* < 0.05 compared with the LPS-stimulated group. (**b**) The nuclear translocation of NF-κB/p65 (green) in cells was analyzed by immunofluorescence. Nuclei were stained with DAPI (blue). Scale bar = 10 μm.

To elucidate the mechanisms through which SME decreases the production of inflammatory mediators, we investigated its effects on both the NF-κB and MAPK signaling pathways in BV2 microglial cells. Initially, we assessed whether SME influences the nuclear translocation of NF-κB. Typically, NF-κB is retained in the cytosol as an inactive complex bound to IκB, comprising the p65 and p50 subunits. Phosphorylation of IκB leads to the disassembly of this complex, enabling NF-κB/p65 to translocate into the nucleus and trigger the transcription of inflammation-related genes [43]. We conducted subcellular fractionation on LPS-stimulated BV2 cells treated with SME. As shown in Figure 4a, SME treatment increased the IκB levels in the cytosol, suggesting reduced IκB degradation. Concurrently, SME significantly reduced nuclear p65 accumulation compared with LPS-treated cells, indicating its effective inhibition of NF-κB nuclear translocation. This finding was further supported by immunofluorescence staining, which revealed that SME inhibited the translocation of p65 from the cytosol to the nucleus in the presence of SME, even under LPS stimulation (Figure 4b). In addition to NF-κB, we also examined the role of the MAPK signaling pathway, another critical mediator in the inflammatory response activated by LPS through phosphorylation events [44]. We assessed the effect of SME on the phosphorylation status of key MAPKs, including p38, p44/42 (Erk1/2), and JNK, in LPS-stimulated BV2 cells. Our results show that SME significantly reduced the phosphorylation of all three MAPKs (Supplementary Figure S1), indicating that SME effectively downregulates MAPK signaling. Overall, these findings suggest that SME achieves its anti-inflammatory effects by blocking both the NF-κB and MAPK signaling pathways, thereby reducing the expression of proinflammatory mediators in LPS-activated microglial cells.

### 2.5. SME Activates the Expression of Nrf2 and HO-1 in LPS-Stimulated Microglial Cells

**Figure 5:**
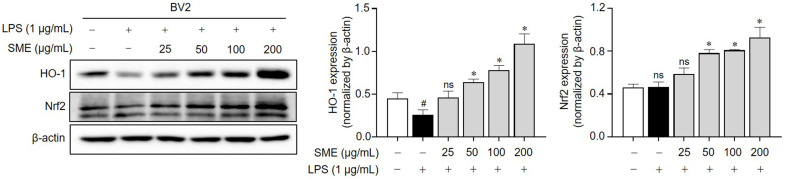
Impact of SME on HO-1 and Nrf2 expression in BV cells stimulated by LPS. BV2 cells were pretreated with SME (25, 50, 100, and 200 μg/mL) for 2 h, followed by stimulation with LPS (1 μg/mL) for 24 h. HO-1 and Nrf2 protein expressions were determined by western blot analysis, with β-actin serving as the loading control. The protein expression was normalized β-actin, and it is presented as a quantitative bar graph. The data are expressed as the mean ± standard error of the mean (SEM) and are based on results from three separate experiments. Group comparisons were conducted using the Mann–Whitney U test for statistical analysis, with significance thresholds set at *p* < 0.05, (^#^) *p* < 0.05 compared with the untreated group, (ns) not significant compared with the untreated group or the LPS-stimulated group, and (*) *p* < 0.05 compared with the LPS-stimulated group.

Previous research established a link between oxidative stress and neuroinflammation [45,46], with ROS generated by microglia under oxidative stress conditions leading to the activation of astrocytes and neighboring microglia, thereby exacerbating the inflammatory response [47]. Given the critical role of the Nrf2/HO-1 pathway in the cellular response to oxidative stress, we investigated the potential of SME to exert antioxidant effects via this pathway in LPS-stimulated BV2 microglial cells. Nrf2 is a crucial transcription factor in the cellular antioxidant defense mechanism, binding to antioxidant response elements (AREs) within the DNA and triggering the expression of genes responsible for antioxidant responses, including HO-1 [48,49]. As shown in Figure 5, our results revealed that SME treatment significantly upregulated the expression of both Nrf2 and HO-1 compared with LPS-stimulated control cells in a dose-dependent manner. These results suggest that the antioxidant effects exhibited by the SME are partially driven by activation of the Nrf2/HO-1 signaling pathway, thereby bolstering defenses against oxidative stress.

### 2.6. SME Exerts Antioxidant Properties in LPS-Stimulated Microglial Cells

**Figure 6:**
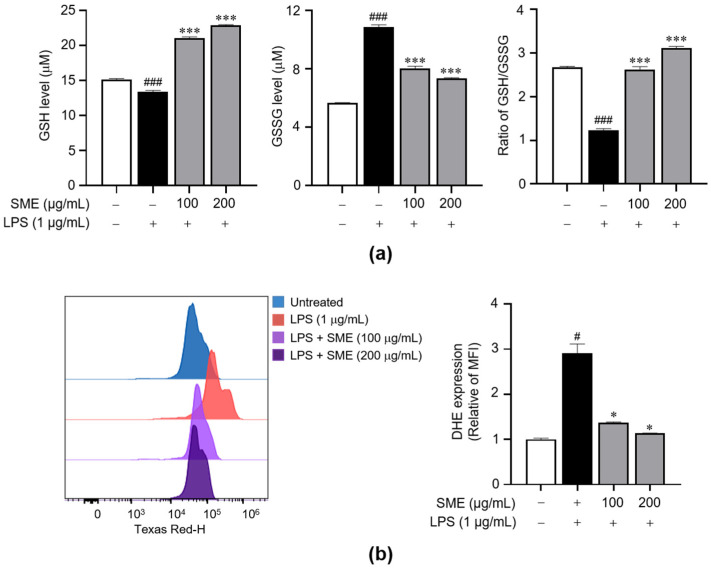
Effects of SME on intracellular ROS and GSH levels in LPS-stimulated BV2 cells. BV2 cells were pretreated with SME (100 and 200 μg/mL) for 2 h, followed by LPS (1 μg/mL) stimulation for 24 h. (**a**) Intracellular ROS levels were quantified by flow cytometry using the DHE fluorescent dye, and they are depicted in the bar graph. (**b**) The total GSH and GSSG and the GSH/GSSG ratio were measured using a GSH assay kit. The data are expressed as the mean ± standard error of the mean (SEM) and are based on results from three separate experiments. Group comparisons were conducted using the Mann–Whitney U test for statistical analysis, with significance thresholds set at *p* < 0.05. The data are presented as the mean ± standard error of the mean (SEM), and they were derived from three separate experiments. Statistical comparisons between groups were performed using the Mann–Whitney U test, with significance thresholds set at *p* < 0.05, (^#^) *p* < 0.05, and (^###^) *p* < 0.001 compared with the untreated group and (*) *p* < 0.05 and (***) *p* < 0.001 compared with the LPS-stimulated group.

To further assess the antioxidant capacity of SME, we employed a DPPH assay to measure its radical scavenging activity. These results indicate that SME exhibited a dose-dependent inhibition of DPPH radicals, achieving effects comparable to quercetin, a standard antioxidant molecule, particularly at a concentration of 200 μg/mL (Supplementary Figure S2). Building on these findings, we proceeded to investigate the antioxidant effects of SME in BV2 microglial cells subjected to LPS-induced oxidative stress by measuring the intracellular levels of GSH and GSSG and the GSH/GSSG ratio. SME pretreatment dose-dependently increased the intracellular GSH levels, improved the GSH/GSSG ratio, and decreased GSSG levels compared with the LPS-treated group (Figure 6a). Furthermore, we evaluated the effect of SME on intracellular ROS levels in LPS-stimulated BV2 cells. Excessive ROS accumulation, which surpasses the cellular antioxidant defense capacity, can result in oxidative stress and inflammation, a process closely regulated by Nrf2-mediated antioxidant enzyme expression [49,50]. Our results demonstrate that SME pretreatment markedly decreased ROS production in these cells (Figure 6b). Collectively, these findings suggest that SME exerts strong antioxidant effects, mitigating oxidative stress in microglial cells.

### 2.7. SME Exhibits Anti-Inflammatory Activity Through an HO-1-Dependent Mechanism

**Figure 7:**
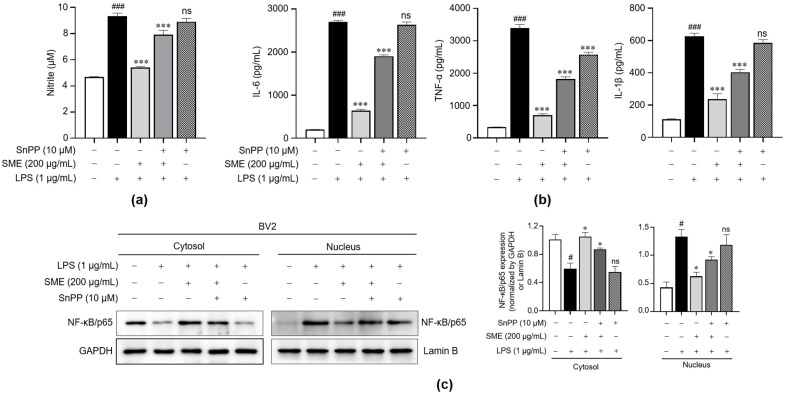
Effects of SME on the inflammatory signaling via an HO-1-dependent mechanism in LPS-stimulated BV2 cells. BV2 cells were pretreated with SME (200 μg/mL) for 2 h with or without SnPP (10 μM), followed by LPS (1 μg/mL) stimulation for 24 h (**a**,**b**) or 15 min (**c**). (**a**) NO levels in the cell supernatant were quantified using the Griess reagent, with NO levels calculated from a standard curve of the nitrite solution concentration. (**b**) The IL-6, TNF-α, IL-1β, and IL-10 secretions were quantified using ELISA. (**c**) NF-κB/p65 and IκB proteins were analyzed in both cytosolic and nuclear fractions via western blot analysis, with GAPDH serving as the loading control for the cytosolic fraction. Meanwhile, lamin B was used as the nuclear loading control. The protein expression levels were normalized to GAPDH and lamin B, and they are depicted in a bar graph. The data are expressed as the mean ± standard error of the mean (SEM) and are based on results from three separate experiments. Group comparisons were conducted using the Mann–Whitney U test for statistical analysis, with significance thresholds set at *p* < 0.05, (^#^) *p* < 0.05, and (^###^) *p* < 0.001 compared with the untreated group and (*) *p* < 0.05, (***) *p* < 0.001, and (ns) not significant compared with the LPS-stimulated group.

To explore the underlying mechanism which links the anti-inflammatory and antioxidant effects of SME, we focused on the Nrf2/HO-1 signaling pathway. Nrf2 is a key regulatory factor known to suppress NF-κB activity, forming a feedback loop which is crucial in controlling inflammation. HO-1, which is activated downstream of Nrf2, plays a pivotal role in this process by significantly modulating inflammatory responses [51]. Previous studies have shown that HO-1 can suppress the nuclear translocation of NF-κB, thereby mitigating inflammation through the action of enzymatic products [52]. In our study, we used Sn-protoporphyrin (SnPP), a specific inhibitor of HO-1, to treat LPS-stimulated BV2 microglial cells, assessing its impact on the NO production and the release of proinflammatory cytokines. As shown in Figure 7a,b, our results demonstrate that blocking HO-1 activity with SnPP significantly reversed the SME-induced suppression of NO production and the release of proinflammatory cytokines, including IL-6, TNF-α, and IL-1β. Additionally, we explored how HO-1 influences the localization of NF-κB within these cells. Notably, SME treatment reduced the nuclear translocation of NF-κB in LPS-stimulated cells without SnPP. However, this inhibitory effect was not observed when HO-1 was downregulated by SnPP (Figure 7c). These observations suggest that the anti-inflammatory effects of SME are closely linked to its ability to inhibit NF-κB activity through the regulation of HO-1 expression in LPS-stimulated BV2 microglial cells.

## 3. Discussion

The anti-inflammatory and antioxidant effects of plant extracts have been widely studied, with numerous reports highlighting their therapeutic potential [53,54,55,56]. The rich history of *S*. *septemtrionalis* in traditional medicine includes reports of various therapeutic effects, such as anti-inflammatory and anti-nociceptive activities, as well as diuretic and neuropharmacological properties [20,21]. However, the molecular signaling mechanisms underlying its anti-inflammatory and antioxidant effects in microglial cells remain poorly understood. Therefore, this study aimed to elucidate the effects of SME on LPS-stimulated BV2 microglial cells, focusing primarily on its impact on neuroinflammation and oxidative stress.

Inflammation plays a critical role in the development of various diseases, especially within the CNS, where it is intricately associated with neurodegenerative disorders such as AD, PD, and MS [57,58,59]. Microglial cells, the primary immune cells in the CNS, play a crucial role in mediating the innate immune response during such inflammatory conditions. These cells respond to pathogen-associated molecular patterns (PAMPs) or damage-associated molecular patterns (DAMPs) by initiating defensive mechanisms, which include the regulation of phagocytosis and the release of proinflammatory molecules [1,60]. When aberrantly activated, microglia can generate elevated levels of proinflammatory mediators, such as IL-1β, IL-6, TNF-α, NO, and ROS, potentially resulting in neuronal damage and cell death [1,5,61]. Our results demonstrate that SME significantly reduces NO production, iNOS and COX-2 expression, and the release of proinflammatory cytokines in LPS-stimulated BV2 microglial cells dose-dependently (Figure 3). Given that the MAPK and NF-κB signaling pathways are pivotal in producing these inflammatory mediators [62], we investigated whether the anti-inflammatory effects of SME are mediated through these pathways. Our findings reveal that SME reduced the phosphorylation of key proteins in the MAPK pathway, including p44/42, JNK, and p38, and effectively inhibited NF-κB translocation to the nucleus (Figure 4 and Supplementary Figure S1).

Oxidative stress is a well-recognized contributor to the pathophysiology of neurodegenerative diseases, where it can promote the aggregation of misfolded proteins, mitochondrial dysfunction, and activation of cell death pathways [63,64]. In this context, oxidative stress also exacerbates neuroinflammation, thereby accelerating neuronal injury and disease progression [10,63]. Therapeutic strategies targeting oxidative stress, such as using antioxidants and compounds which enhance mitochondrial function, are being actively explored [65,66]. The involvement of ROS in the progression of neurodegenerative diseases is well documented [67], and LPS has been experimentally confirmed to induce oxidative stress, elevating ROS levels in microglial cells [68]. Previous studies have demonstrated that certain botanical extracts exert anti-inflammatory effects in microglial cells by suppressing the MAPK and NF-κB pathways and activating the Nrf2 pathway and ARE [69,70,71,72]. Excessive ROS has been found to exacerbate the expression of inflammatory mediators such as iNOS and COX-2, processes which the Nrf2/HO-1 axis may modulate [73]. In our study, SME significantly upregulated the expression of HO-1 and Nrf2 (Figure 5), increased intracellular GSH levels, and reduced ROS levels (Figure 6), indicating its potent antioxidant properties in LPS-stimulated BV2 microglial cells.

Additionally, we explored the involvement of the Nrf2/HO-1 signaling pathway in relation to oxidative stress and inflammation. Our findings align with previous research, highlighting the Nrf2/HO-1 axis as a crucial defense mechanism against oxidative stress [74,75,76]. For instance, Luo et al. reported that SnPP treatment effectively inhibited the anti-inflammatory effects of celastrol, particularly its ability to suppress the expression of iNOS and COX-2, and the proinflammatory cytokines IL-6, TNF-α, and IL-1β [77]. Similarly, in our study, blocking HO-1 activity with the specific inhibitor SnPP reversed the suppressive effects of SME on the production of inflammatory mediators, including NO, and pro-inflammatory cytokines (IL-6, TNF-α, and IL-1β) (Figure 7a,b). This supports our hypothesis that the Nrf2/HO-1 pathway partially mediates the anti-inflammatory effects of SME. Moreover, Kim et al. demonstrated that SnPP treatment abolished the effect of narchinol B in reducing nuclear p65 levels following LPS treatment [78]. Our results also revealed that the inhibitory effect of SME on NF-κB nuclear translocation was significantly counteracted by SnPP (Figure 7c), further underscoring the pivotal role of HO-1 in modulating NF-κB activity under the condition of LPS-induced oxidative stress in microglial cells. Together, these findings highlight the Nrf2/HO-1 signaling pathway as a promising therapeutic target for controlling neuroinflammation.

In addition, the pharmacological effects of plant extracts are intricately related to the diverse phytochemicals they contain, each contributing to the overall therapeutic outcome. In the present study, UPLC-Q-TOF-MS analysis identified emodin (compound 16), isobrucein A (compound 17), and 1β-hydroxy euscaphic acid (compound 19) as the major constituents in SME (Figure 1 and Table 1). Emodin in particular is well recognized for its strong antioxidant and anti-inflammatory properties, as substantiated by numerous studies [79,80,81,82]. Emodin has been shown to alleviate oxidative stress and inflammation through various mechanisms, such as modulating the NF-κB and NLRP3 inflammasome pathways to protect against neurological damage [80] and inhibiting proinflammatory cytokines, such as IL-6 and IL-1β, by influencing the NF-κB and MAPK signaling pathways [79]. Additionally, emodin-mediated activation of the Nrf2/Keap1 pathway further emphasizes its role in mitigating oxidative damage [82] and reducing inflammation markers in severe acute pancreatitis models [81]. Furthermore, studies have shown that emodin, which is abundant in the roots and rhizomes of *Polygonum cuspidatum*, reduces NO production and proinflammatory cytokines in LPS-activated Raw264.7 cells, thereby exerting significant anti-inflammatory effects [83]. Similarly, *Radix Polygoni Multiflori* is derived from the roots of *Polygonum multiflorum* Thunb. and is rich in emodin. Moreover, emodin has been shown to alleviate metabolic inflammation associated with nonalcoholic fatty liver disease (NAFLD) in mouse hepatocytes by modulating MAPK signaling pathways [84]. These findings suggest that emodin is a key contributor to the antioxidant and anti-inflammatory activities observed in SMEs. Conversely, the contributions of isobrucein A and 1β-hydroxy euscaphic acid to these effects are poorly understood. Additionally, despite 1β-hydroxy euscaphic acid being documented for its hepatoprotective effects, particularly in reducing intracellular enzyme leakage [85], there is limited evidence of its antioxidant or anti-inflammatory capabilities. Similarly, no studies have yet confirmed such properties for isobrucein A. Therefore, to gain a more comprehensive understanding of the pharmacological effects of SME, further investigation into the individual and synergistic activities of isobrucein A and 1β-hydroxy euscaphic acid is essential. This research could uncover additional mechanisms through which these compounds contribute to the overall therapeutic profile of SME.

## 4. Materials and Methods

### 4.1. Plant Extract

*S*. *septemtrionalis* (Viv.) H.S. Irwin & Barneby, commonly known as the arsenic bush, was sourced from the Da Chais community of the Lac Duong district in Lam Dong, Vietnam (N 12°10′29.9″, E 108°40′33.9″). Dr. Tran The Bach collected and identified plant samples at the Institute of Ecology and Biological Resources (Hanoi, Vietnam). Voucher specimens recorded as VK 4880 were deposited at the herbarium of the Korea Research Institute of Bioscience and Biotechnology (Daejeon, Republic of Korea). *S*. *septemtrionalis* leaves, branches, and fruits (80 g) were mixed with 99.9% MeOH (1 L) and sonicated several times at room temperature (RT) over three days. The resulting MeOH extracts (6.11 g) were filtered and evaporated at 40 °C under reduced pressure to produce crude extracts.

### 4.2. Ultra-HPLC-Quadrupole Time-of-Flight Mass Spectrometry (UPLC-Q-TOF-MS) Analysis

This study diluted the extract (1 mg) in methanol (1 mL) and centrifuged it at 12,000 rpm and RT for 10 min. Then, the mixture was filtered through a microporous membrane (0.22 μm) and subjected to qualitative analysis. Chromatographic separation and mass spectrometric detection were performed simultaneously using an integrated UPLC-Q-TOF-MS system (Waters, Milford, MA, USA). Chromatographic separation was performed using an ACQUITY UPLC^®^ system fitted with a BEH C18 column (2.1 × 100 mm, 1.7 µm; Waters). The mobile phases employed (1) water containing 0.1% formic acid and (2) acetonitrile with 0.1% formic acid. The gradient elution program was as follows: 0–1 min = 5% B; 1–22.3 min = 5%–100% B; 22.3–22.4 min = 5% B; and 22.4–25 min = 5% B. The flow rate was set to 0.4 mL/min. The column temperature was maintained at 35 °C, and the injection volume was 2 µL. The eluted compounds were directly introduced into an SYNAPT G2-Si mass spectrometer (Waters, Milford, MA, USA) for mass spectrometric analysis. The mass spectrometer was set to operate in negative electrospray ionization (ESI) mode with the following parameters: capillary voltage = 1 kV; sampling cone voltage = 20 V; source temperature = 120 °C; desolvation temperature = 450 °C; and desolvation gas flow rate = 800 L/h. The collision energy was set at 4 eV, and data were acquired over a mass range of 50–1200 *m*/*z*.

### 4.3. Cell Culture

BV2 murine microglial cells were purchased from AcceGen (#ABC-TC212S, Fairfield, NJ, USA). The BV2 cells were cultured in Dulbecco’s Modified Eagle’s Medium (DMEM) (#LM0001-05, WELGENE Inc., Gyeongsan, Republic of Korea) supplemented with 10% fetal bovine serum (FBS) (#16000044, Thermo Fisher Scientific Inc., Waltham, MA, USA) and 1% penicillin-streptomycin (#30-002-CI, Corning Inc., Corning, NY, USA) in an incubator at 37 °C and containing 5% CO_2_.

### 4.4. Cell Viability Test

Cell viability was evaluated using an EZ-Cytox assay kit based on the WST (#EZ-1000, DoGen Bio, Seoul, Republic of Korea), following the instructions provided by the manufacturer. For the experiments, cells were plated on a 96-well plate (2 × 10^4^ cells/well) in triplicate and incubated with SME (25, 50, 100, and 200 µg/mL) for 24 h at 37 °C in a 5% CO_2_ atmosphere. The WST reagent was added post incubation, and the cells were further incubated at 37 °C in a 5% CO_2_ atmosphere for 30 min. The absorbance of the samples was measured at 450 nm using a Synergy HTX microplate reader (BioTek Instruments, Winooski, VT, USA). The control cells received only the conditioned media without LPS or SME treatment.

### 4.5. Cytotoxicity Assay

Cytotoxicity was determined by releasing LDH from dead cells, according to the manufacturer’s protocol, using an LDH assay kit (#DG-LDH500, DoGen Bio). In detail, the cells were seeded (2 × 10^4^ cells/96-well plate) in triplicate and pretreated with SME (25, 50, 100, and 200 µg/mL) for 2 h. After pretreatment, the cells were stimulated with lipopolysaccharide (LPS, 1 µg/mL) (#L4516, Sigma-Aldrich, St. Louis, MO, USA) for 24 h. The supernatants from the cell cultures were then collected and distributed into 96-well plates in triplicate. The plates were treated with LDH reagent and kept in the dark at RT for 30 min. The absorbance of the samples was then measured at 450 nm using a Synergy HTX microplate reader (BioTek Instruments). The calculation used for cytotoxicity has been described previously [86].

### 4.6. Western Blot Analysis

The cells were pretreated with SME (25, 50, 100, and 200 µg/mL) for 2 h, followed by stimulation with LPS (1 µg/mL) for 15 min or 24 h. After the treatment period, the cells were lysed with radioimmunoprecipitation assay buffer (RIPA) (#RC2002-050-00, Biosesang, Seongnam, Republic of Korea) supplemented with a protease inhibitor cocktail (#P8340) and phosphatase inhibitor cocktail II (#P5726) and III (#P0044) (Sigma-Aldrich). The cell lysates were then homogenized using a sonicator (Sonics & Materials Inc., Newtown, CT, USA). The denatured proteins were resolved using sodium dodecyl sulfate-polyacrylamide gel electrophoresis (SDS_PAGE) and transferred onto nitrocellulose membranes. The membranes were subsequently blocked using 5% skimmed milk (#SKI500, LPS Solution, Daejeon, Republic of Korea) prepared in phosphate-buffered saline (PBS) containing 0.05% Tween 20 (#TW2001, LPS Solution). Then, the membranes were incubated with anti-iNOS (#610332, BD Biosciences, San Diego, CA, USA), anti-COX-2 (#sc-166475), anti-p38 (#sc-7972), anti-p44/42 (Erk1/2, #sc-514302), anti-HO-1 (#sc-390991), and anti-β-actin (#sc-47778) (Santa Cruz Biotechnology Inc., Dallas, TX, USA) as well as anti-SAPK/JNK (#9252), anti-phospho-SAPK/JNK (#9251), anti-phospho-p38 (#9211), anti-phospho-p44/42 (#9101), and anti-Nrf2 (#12721) (Cell Signaling Technology, Danvers, MA, USA) antibodies at 4 °C overnight. Further, each membrane was rinsed three times with Tris-buffered saline (TBS) containing 0.05% Tween 20 and then incubated with the peroxidase-conjugated anti-mouse (#7076) and anti-rabbit (#7074) antibodies (Cell Signaling Technology) at RT for 1 h. The protein bands were developed using an enhanced chemiluminescence (ECL) solution and visualized with a ChemiDoc Imaging System (Amersham Imager 680, Cytiva, MA, USA). Protein expression levels were quantified using ImageQuant TL software (version 8.2, GE Healthcare Inc., Sunnyvale, CA, USA).

### 4.7. Measurement of Intracellular Reactive Oxygen Species (ROS)

Intracellular ROS levels were quantified using dihydroethidium (DHE) (D23107, Molecular Probes, Eugene, OR, USA). Briefly, BV2 cells were seeded (1 × 10^6^ cells/6-well plate) and pretreated with SME (100 and 200 µg/mL) for 2 h, followed by stimulation with LPS (1 µg/mL) for 24 h. The cells were subsequently stained with 5 μM DHE at 37 °C in the dark for 30 min. Following a wash with 1 × PBS, DHE fluorescence was detected using a NovoCyte Flow Cytometer (Agilent Technologies, Santa Clara, CA, USA) and analyzed with FlowJo software (version 10.9.0, BD Biosciences). A total of 20,000 events were recorded for each sample, and the data are presented as the mean fluorescence intensity, represented by both spectral and quantitative bar graphs. 

### 4.8. Glutathione (GSH) Assay

The GSH assay was performed following a previously reported protocol [86]. Briefly, cells were plated in 6-well plates (1 × 10^6^ cells/well) and pretreated with SME (100 and 200 μg/mL) for 2 h. Following pretreatment, the cells were treated with LPS (1 μg/mL) for 10 min, rinsed with cold PBS, and then harvested via centrifugation. The cell pellet was disrupted by sonication in cold MES buffer (50 mM) (from the GSH assay kit (#703002; Cayman Chemical Company, Ann Arbor, MI, USA)), followed by centrifugation at 10,000× *g* and 4 °C for 15 minutes. The supernatant was then transferred to a new tube, and 2-vinylpyridine (1 M) (#13229-2; Sigma-Aldrich) was added at a volume equivalent to 10% of the supernatant. The prepared samples and standards were placed into 96-well plates, and the assay cocktail mixture (from the GSH assay kit) was added to each well at four times the sample volume. The plate was placed on a rotary shaker and incubated in the dark for 25 min, followed by measuring the absorbance at 410 nm using a Synergy HTX microplate reader (BioTek Instruments). The GSH and GSSG and their ratio were calculated and presented as a bar graph.

### 4.9. 2,2′-Diphenyl-1-Picrylhydrazyl Radical (DPPH) Assay

The DPPH assay was conducted as previously described [86]. Briefly, a high-concentration stock solution of SME (100 mg/mL) and quercetin (100 mg/mL) was prepared using a DMSO solution. Then, the SME was serially diluted with 100% methanol to each concentration (12.5, 25, 50, 100, and 200 μg/mL). Quercetin (#Q4951, Sigma-Aldrich, St. Louis, MO, USA) was diluted to 10 μg/mL in 100% methanol. Subsequently, 100 μL of each diluted sample was added to a 96-well plate, followed by 100 μL of DPPH solution (0.2 mM). The mixture was incubated at 37 °C in an incubator for 30 min. After incubation, the absorbance was recorded at 517 nm using a Synergy H1 Hybrid microplate reader. The DPPH inhibition percentage was calculated by comparing the absorbance of the samples to that of the negative control (100% methanol solution). Quercetin was used as a positive control.

### 4.10. Measurement of NO Contents

The cells were treated with varying SME concentrations (12.5, 25, 50, 100, and 200 µg/mL) for 2 h, followed by stimulation with LPS (1 µg/mL) for 24 h. Following incubation, the cell culture supernatants were gathered and combined with Griess reagent, composed of 1% sulfanilamide (#S9251), 0.1% N-1-naphthylenediamine dihydrochloride (#N9125) (Sigma-Aldrich), and 2.5% phosphoric acid (#1.00573.1000, Merck Millipore, Burlington, MA, USA), and then incubated at RT for 5 min. The absorbance of each sample was read at 540 nm using a Synergy HTX microplate reader (BioTek Instruments). NO production was quantified by comparing the absorbance values to a standard curve generated using a nitrite standard solution.

### 4.11. Enzyme-Linked Immunosorbent Assay (ELISA)

The cells were pretreated with varying SME concentrations (25, 50, 100, and 200 µg/mL) for 2 h, followed by stimulation with LPS (1 µg/mL) for 24 h. After the treatment, the culture supernatants were collected and analyzed for their IL-6, IL-1β, TNF-α, and IL-10 levels using ELISA. In detail, 96-well plates were coated with purified anti-IL-6 (#554400), anti-IL-10 (#551215) (BD Bioscience, San Jose, CA, USA), anti-IL-1β (#14-7012-85), and anti-TNF-α (#14-7423-85) (Thermo Fisher Scientific Inc.) antibodies and incubated at 4 °C overnight. Following incubation, the plates were washed with PBS containing 0.05% Tween 20 and blocked with 1% bovine serum albumin (BSA) in PBS at RT for 1 h. The supernatant samples were added to the wells and incubated at RT for 2 h. After washing, detection antibodies were applied and incubated at RT for 1 h. Streptavidin-conjugated horseradish peroxidase (HRP) (DY998, R&D Systems, Minneapolis, NE, USA) was added to each well and incubated in the dark for 30 min. After washing, the reaction was developed by adding TMB (3, 3′, 5, 5′-tetramethylbenzidine) substrate buffer (#555214, BD Biosciences) and incubating at RT for 20 min. Absorbance was measured at 450 nm using a Synergy HTX microplate reader (BioTek Instruments).

### 4.12. Nucleus and Cytosol Fractionation

Nucleus and cytosol fractionation was conducted using the NE-PER Nuclear and Cytoplasmic Extraction Reagents kit (#78833, Thermo Fisher Scientific Inc.) per the manufacturer’s instructions. Briefly, the cells were treated with SME (100 and 200 µg/mL) for 2 h before stimulation with LPS (1 µg/mL) for 15 min. Following this incubation period, the cells were harvested by centrifugation at 500 g at 4 °C for 5 min, and the supernatants were gently removed. The cell pellets were then lysed using the provided lysis solution, followed by centrifugation to obtain the cytoplasmic extract. Subsequently, the remaining pellets were resuspended in nuclear lysis solution and centrifuged at high speed to isolate the nuclear extract. The supernatants with the nuclear extract were moved to a new, pre-cooled tube. These extracts were then denatured and subjected to western blot analysis using specific antibodies: anti-Lamin B1 (nuclear marker) (#sc-377000), anti-GAPDH (cytosol marker) (#sc-365062), anti-NF-κB p65 (#sc-8008) (Santa Cruz Biotechnology Inc.), and anti-IκB (#9242, Cell Signaling Technology).

### 4.13. Immunofluorescence

The cells were pretreated with SME at 200 µg/mL for 2 hours and then stimulated with LPS (1 µg/mL) for 15 min. Post treatment, the cells were rinsed with PBS and fixed with 4% PFA (paraformaldehyde) solution (#PC2031-050-00, Biosesang) at RT for 10 min. Following fixation, permeabilization was performed using 0.1% Triton X-100 at RT for 10 min. The cells were subsequently incubated with 1% BSA in PBS at RT for 1 h for blocking. After washing, the cells were exposed to the anti-NF-κB antibody and incubated at 4 °C overnight. The next day, the cells were incubated with secondary antibodies in the dark for 1 h. Nuclei were stained with 4′, 6-diamidino-2-phenylindole (DAPI) at RT for 1 h. Finally, the cells were mounted onto slide glasses using a mounting solution (#S36936, Thermo Fisher Scientific Inc.), and fluorescent images were acquired using a confocal microscope (Model; Nikon AX R, Nikon Instruments, Tokyo, Japan).

### 4.14. Statistical Analysis

Data analysis was performed using Prism 8.0 software (GraphPad Inc., San Diego, CA, USA). The results are derived from three separate experiments, and they are expressed as the mean ± standard error of the mean (SEM). Group differences were evaluated using the nonparametric Mann–Whitney U test. A *p* value <0.05 was considered statistically significant.

## 5. Conclusions

**Figure 8:**
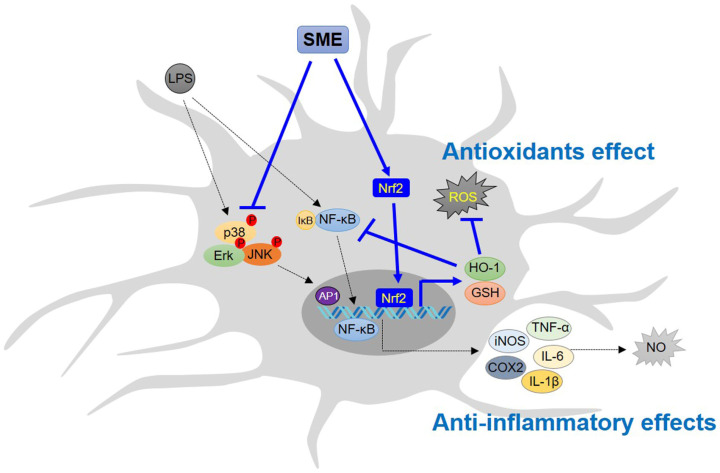
Molecular signaling pathway underlying the antioxidant and anti-inflammatory effects of SME in LPS-stimulated microglial cells. LPS and other stressors induce neuroinflammation by activating the NF-κB and MAPK signaling pathways [87,88,89]. This study demonstrated that SME exerted its antioxidant effects by activating the Nrf2/HO-1 signaling pathway. In contrast, its anti-inflammatory effect was achieved by inhibiting the NF-κB and MAPK signaling pathways in LPS-stimulated BV2 cells. Additionally, treatment with SnPP, a specific HO-1 inhibitor, reversed the suppression of inflammatory mediators such as NO and proinflammatory cytokine in LPS-stimulated BV2 cells. Notably, when SnPP inhibited HO-1 expression in the LPS-stimulated cells, SME treatment failed to inhibit NF-κB nuclear translocation in these cells. In summary, SME exhibited both antioxidant and anti-inflammatory effects on the LPS-activated microglial cells, with its antioxidant effect partially contributing to its anti-inflammatory activity.

This study elucidated the molecular mechanisms through which SME exerts anti-inflammatory and antioxidant properties in BV2 microglial cells. Our findings indicate that SME markedly suppressed the production of inflammatory mediators by inhibiting the MAPK and NF-κB signaling pathways while concurrently upregulating the expression of Nrf2 and HO-1 proteins, demonstrating significant antioxidant properties. Notably, SME activates Nrf2, which leads to increased expression of its key target gene HO-1, thereby inhibiting NF-κB nuclear translocation and regulating the anti-inflammatory response (Figure 8). 

However, this study did not evaluate the direct effects of SME on an animal model of inflammatory disease or its long-term administration, and the mechanistic investigation lacked sufficient experiments involving Nrf2 inhibition. Therefore, further research utilizing in vivo models and targeted Nrf2 inhibition studies is necessary to define the effects of SME and its underlying mechanisms more precisely. Despite these limitations, our robust in vitro data suggest that SME is a promising candidate for regulating neuroinflammation and may hold therapeutic potential for diseases characterized by excessive inflammatory responses.

## Figures and Tables

**Table 1:** Identification of compounds in the SME using UPLC-Q-TOF-MS spectrometry analysis.

#	Observed RT (min)	Neutral Mass (Da)	Observed Neutral Mass (Da)	Observed (*m/z*)	Adducts	Component Name
1	0.54	132.05349	132.0532	131.0459	−H	Asparagine (TML)
2	0.59	150.05282	150.0524	195.0506	+HCOO	Pentose (TML)
3	4.27	696.19016	696.1889	741.1871	+HCOO	Malvidin-3-O-(6-O-acetyl-β-D-glucopyranoside)-5-O-β-D-glucopyranoside [30]
4	4.41	394.12638	394.126	393.1187	−H	Aloeresin [31]
5	4.54	610.15338	610.1532	609.146	−H	Rutin [32]
6	4.76	756.21129	756.2114	755.2041	−H, +Cl	Kaempferol-3-rutinoside-7-glucoside (TML)
7	4.85	594.15847	594.1589	593.1516	−H	Genistein-7,4′-di-O-β-D-glucoside [33]
8	5.03	286.04774	286.0469	285.0396	−H	Kaempferol [34]
9	5.12	624.16903	624.1693	623.1621	−H	Complanatuside [35]
10	5.19	270.05282	270.0523	269.045	−H	Rubilactone [36]
11	5.83	684.26294	684.266	683.2587	−H	Yadanzioside A (TML)
12	5.97	764.39831	764.4041	763.35513	−H	Cynanoside Q2 (TML)
13	6.35	666.39791	666.3975	665.3902	−H, +HCOO	Sericoside [37]
14	6.45	554.19994	554.1981	589.1675	+Cl	Osthenol-7-O-β-D-gentiobioside (TML)
15	6.64	504.34509	504.3459	503.3386	−H	Platyconic acid C (TML)
16	6.8	270.05282	270.0528	269.0455	−H	Emodin [22]
17	7.02	522.21011	522.2154	567.2136	+HCOO	Isobrucein A (TML)
18	7.19	270.05282	270.0524	269.0451	−H	Genistein [38]
19	8.18	504.34509	504.3455	503.3382	−H	1β-hydroxy euscaphic acid (TML)
20	8.41	506.36074	506.3612	505.3539	−H	13,17-epoxy alisol A (TML)
21	8.68	518.32435	518.324	517.3167	−H	Picfeltarraegenin VII (TML)
22	9.06	582.11621	582.1159	581.1086	−H	5′-methoxy-bilobetin (TML)
23	9.16	504.34509	504.3447	503.3374	−H	Brahmic acid (TML)
24	9.92	570.17373	570.1743	569.167	−H	2″-O-feruloylaloesin (TML)
25	11.2	226.06299	226.0634	225.0561	−H	Oroselone (TML)
26	12.02	566.1213	566.1208	565.1135	−H	Isoginkgetin [39]
27	12.95	542.39712	542.3955	577.3649	+Cl	Methyl pachymate (TML)
28	15.64	510.26175	510.2639	555.2621	+HCOO	Ganoderenic acid F (TML)
29	16.09	410.35487	410.3553	455.3535	+HCOO	Stigmasterone (TML)
30	18.17	310.32357	310.3235	355.3217	+HCOO	n-henicosanal (TML)
31	19	206.16707	206.1669	205.1596	−H	Longicamphenylone [40]
32	19.57	384.23006	384.2297	383.2225	−H	Resibufogenin [41]

TML = traditional medicine library.

## Data Availability

The data presented in this study are available in the article and Supplementary Materials.

## Supplementary Materials

The following supporting information can be downloaded at https://www.mdpi.com/article/10.3390/ijms26051932/s1.

## Abbreviations

The following abbreviations are used in this manuscript:

SME	methanol extract of *Senna septemtrionalis* (Viv.) H.S. Irwin & Barneby
LPS	lipopolysaccharide
NO	nitric oxide
TNF	tumor necrosis factor
IL	interleukin
MAPK	mitogen-activated protein kinase
NF-κB	nuclear factor kappa-chain-enhancer of activated B cells
IκB	inhibitor κB
Nrf2	nuclear factor erythroid 2-related factor 2
HO-1	heme oxygenase-1
ROS	reactive oxygen species
SnPP	Sn-protoporphyrin
CNS	central nervous system
BBB	blood–brain barrier
AD	Alzheimer’s disease
PD	Parkinson’s disease
MS	multiple sclerosis
WST	water-soluble tetrazolium salt
LDH	lactate dehydrogenase
iNOS	inducible nitric oxide synthase
COX-2	cyclooxygenase-2
PGE2	prostaglandin E2
DHE	dihydroethidium
GSH	glutathione
GSSG	glutathione disulfide
DPPH	2,2′-diphenyl-1-picrylhydrazyl radical
TMB	3, 3′, 5, 5′-tetramethylbenzidine
PFA	paraformaldehyde
DAPI	4′, 6-diamidino-2-phenylindole
SEM	standard error of the mean
TIC	total ion chromatogram
TML	traditional medicine library
ARE	antioxidant response elements
PAMP	pathogen-associated molecular pattern
DAMP	damage-associated molecular pattern
Erk	extracellular signal-regulated kinase
JNK	c-Jun N-terminal kinase
AP-1	activator protein-1
