# Supplementary material for: Antioxidant and Anti-Inflammatory Activities of Methanol Extract of Senna septemtrionalis (Viv.) H.S. Irwin & Barneby Through Nrf2/HO-1-Mediated Inhibition of NF-κB Signaling in LPS-Stimulated Mouse Microglial Cells
Source: Int J Mol Sci. 2025 Feb 24;26(5):1932. doi: 10.3390/ijms26051932 (PMC11900505; doi:10.3390/ijms26051932)
Supplement: Supplementary file 1 [file ijms-26-01932-s001.zip › ijms-3418158-supplementary.pdf]

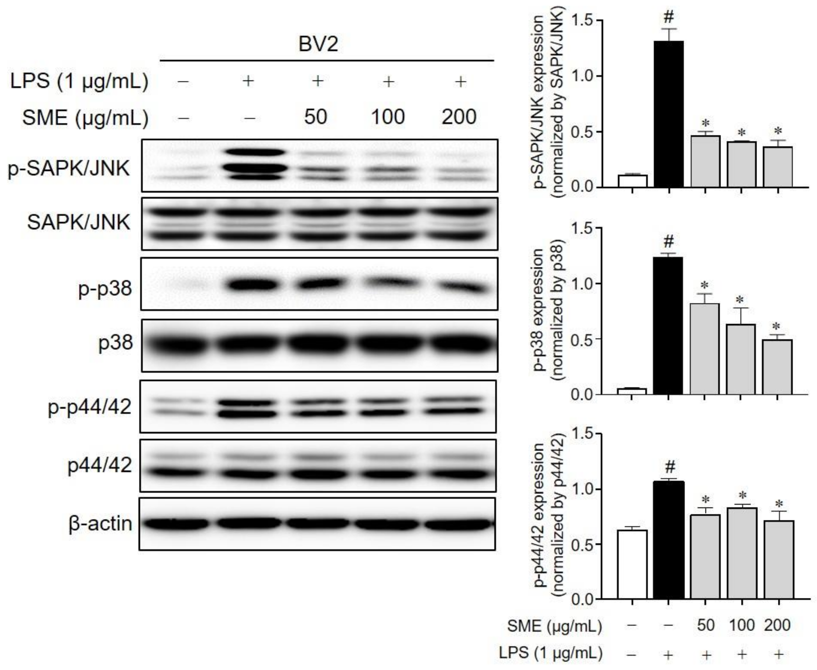

Supplementary Figure S1. SME suppresses MAPK phosphorylation in LPS-stimulated BV2 cells. BV2 cells were pretreated with SME (50, 100, and 200 μg/mL) for 2hr, followed by stimulation with LPS (1 μg/mL) for 15 min. Phosphorylation and total protein levels of the MAPK signaling pathway (JNK, p44/42, and p38) were analyzed via western blot, with β-actin serving as the loading control. Phosphorylated forms of SAPK/JNK, p44/42, and p38 were normalized to their respective total protein levels. The data are expressed as the mean ± standard error of the mean (SEM) and are based on results from three separate experiments. Group comparisons were conducted using the Mann-Whitney *U* test for statistical analysis, with significance thresholds set at p-value < 0.05. #p < 0.05 compared to the Untreated group; *p <0.05 compared to the LPS-stimulated group. MAPK, mitogen-activated protein kinase; SAPK/JNK, stress-activated protein kinase/c-Jun N-terminal kinase; p-, phosphorylated.

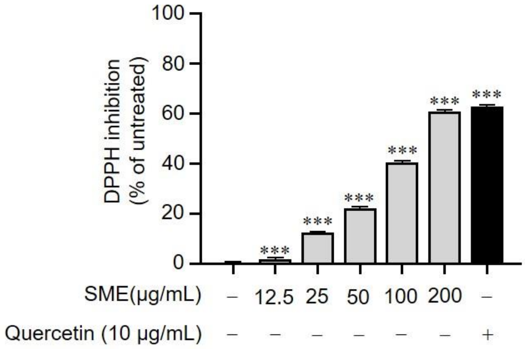

Supplementary Figure S2. SME exhibits an inhibitory effect on the free radical DPPH. DPPH radical scavenging capacity of SME was evaluated at concentrations of 12.5, 25, 50, 100, and 200 μg/mL, with Quercetin (10 μg/mL) serving as a reference compound. The results are presented as the percentage of DPPH inhibition compared to the untreated group. The data are expressed as the mean ± standard error of the mean (SEM) and are based on results from three separate experiments. Group comparisons were conducted using the Mann-Whitney *U* test for statistical analysis, with significance thresholds set at p-value < 0.05. ***p <0.001 compared to the Untreated group. DPPH, 2,2-diphenyl-1-picrylhydrazyl.
